# Polymorphisms of *STS* gene and *SULT2A1* gene and neurosteroid levels in Han Chinese boys with attention-deficit/hyperactivity disorder: an exploratory investigation

**DOI:** 10.1038/srep45595

**Published:** 2017-04-03

**Authors:** Liang-Jen Wang, Wen-Ching Chan, Miao-Chun Chou, Wen-Jiun Chou, Min-Jing Lee, Sheng-Yu Lee, Pao-Yen Lin, Yi-Hsin Yang, Cheng-Fang Yen

**Affiliations:** 1 Department of Child and Adolescent Psychiatry, Kaohsiung Chang Gung Memorial Hospital and Chang Gung University College of Medicine, Kaohsiung, Taiwan; 2 Department of Psychiatry, School of Medicine, and Graduate Institute of Medicine, College of Medicine, Kaohsiung Medical University, Kaohsiung, Taiwan; 3 Center for Research Informatics, The University of Chicago, Chicago, Illinois, 60637, United States of America; 4 Department of Psychiatry, Kaohsiung Veterans General Hospital, Kaohsiung, Taiwan; 5 Department of Psychiatry, College of Medicine and Hospital, National Cheng Kung University, Tainan, Taiwan; 6 Department of Psychiatry, Kaohsiung Chang Gung Memorial Hospital and Chang Gung University College of Medicine, Kaohsiung, Taiwan; 7 School of Pharmacy, Kaohsiung Medical University Hospital, Kaohsiung, Taiwan; 8 School of Pharmacy, Kaohsiung Medical University, Kaohsiung, Taiwan.

## Abstract

This study examined the relationships among polymorphisms of the *STS* gene and *SULT2A1* gene, dehydroepiandrosterone (DHEA) and its sulfated form (DHEA-S), and characteristics of attention-deficit/hyperactivity disorder (ADHD). We used cheek swabs to obtain the genomic DNA of 200 ADHD male probands (mean age: 8.7 years), 192 patients’ mothers and 157 patients’ fathers. Three SNPs in the *STS* gene (rs6639786, rs2270112, and rs17268988) and one SNP in the *SULT2A1* gene (rs182420) were genotyped. Saliva samples were collected from the ADHD patients to analyze DHEA and DHEA-S levels. The behavioral symptoms were evaluated with the Swanson, Nolan, and Pelham, and Version IV Scale for ADHD (SNAP-IV), and the neuropsychological function was assessed using the Conners’ Continuous Performance Tests (CPT). We found the C allele of rs2270112 within the *STS* gene to be over-transmitted in males with ADHD. Polymorphisms of rs182420 within the *SULT2A1* gene were not associated with ADHD. In addition, the C allele carriers of rs2270112 demonstrated significantly higher DHEA-S levels than the G allele carriers. Levels of DHEA were positively correlated with attention as measured by the CPT. These findings support a potential role in the underlying biological pathogenesis of ADHD with regard to *STS* polymorphisms and neurosteroid levels.

Attention deficit/hyperactivity disorder (ADHD) is a common neurodevelopmental disorder that affects approximately 3–10% school-age children around the world^1^,^2^. The core symptoms of ADHD are inattention, hyperactivity, and impulsivity, all of which have a significantly negative impact on patients’ functioning^3^. Although the underlying pathophysiological mechanisms of ADHD are complex and multidimensional, neurosteroids have been connected with ADHD susceptibility^4^. Dehydroepiandrosterone (DHEA) and its sulfated form (DHEA-S) are important neurosteroid substrates that are critical for various physiological processes and involved in several neuropsychiatric diseases^5^. DHEA(S) may facilitate neurite extension and neural cell proliferation and is thus important for regulating neurodevelopment^6^,^7^. Furthermore, DHEA(S) exerts stimulatory or antagonistic effects at gamma-aminobutyric acid (GABA_A_) receptors and facilitates N-methyl-D-aspartate (NMDA) activity^8^. DHEA and DHEA-S levels in blood are inversely related to the severity of hyperactivity/impulsivity symptoms in young male with ADHD^9^. We previously reported that salivary DHEA levels were significantly lower in ADHD patients than those in healthy controls^10^. Furthermore, DHEA(S) levels in ADHD patients increased significantly while they underwent medication treatment^11^,^12^,^13^. Therefore, DHEA(S) is considered a potential biomarker for ADHD^14^,^15^.

The sex discrepancy in many ADHD characteristics is significant. The prevalence of ADHD is significantly higher in males (the ratios of boys to girls range from 2:1 to 9:1)^1^,^2^. Previous research into ADHD has established a strong genetic component to the phenotype^16^. The significantly higher incidence of males suffering from the disorder suggests that some susceptibility genes may lie on the X chromosome^17^. The steroid sulfatase (*STS*) gene, located at Xp22.32, encodes a multi-pass membrane protein of the endoplasmic reticulum^18^. The protein is the property of sulfatase family, and is involved in the pathway that converts neurosteroid sulfates into their free forms (e.g., DHEA-S to DHEA)^19^,^20^. Several animal studies have revealed that the 39,X(Y)*O mice with deletion of the *STS* gene exhibit abnormal behaviors and cognitive functions that are relevant to the inattention and hyperactivity that many ADHD patients experience^21^,^22^. The ADHD-like features seemed to be correlated with the dysregulation of steroid hormone levels and neurotransmitters^23^,^24^, and improvement may be triggered through administration with DHEA-S^25^.

In humans, the *STS* gene is correlated with androgen synthesis and metabolism^26^, while *STS* deficiency has been proposed as a risk factor for ADHD^19^,^27^. Chatterjee *et al*.^28^ reported a link between X-linked ichthyosis (*STS* deficiency) and ADHD diagnosis/symptoms. Brookes *et al*.^29^ performed the transmission disequilibrium test (TDT) analysis, which produced two single nucleotide polymorphisms (SNPs) that were overtransmitted in patients with ADHD (C allele at rs2770112 and G allele at rs12861247). Subsequently, five more SNPs on the *STS* gene (C allele at rs1473666, T allele at rs6639786, T allele at rs5934769, G allele at rs5934770, and G allele at rs17335568), as well as an allele specific haplotype of 12 SNPs, were found to be significantly associated with ADHD^30^. Furthermore, Stergiakouli *et al*.^31^ reported that G allele at rs17268988 was related to inattentive symptoms, while polymorphisms within *STS* were related to cognitive performance among ADHD males. However, said association between *STS* polymorphisms and ADHD has not yet been examined in a non-Caucasian population.

The *SULT2A1* gene, located at 19q13.3, encodes a member of the sulfotransferase family^32^, and functions as a catalyst for the sulfate conjugation of DHEA and other steroids^33^. In this way, the *SULT2A1* gene may participate in ADHD pathophysiology^19^,^27^. Garcia-Anguita *et al*.^34^ indicated that the polymorphisms in the *SULT2A1* gene (rs2637125 and rs182420) may affect DHEA-S concentration in post-pubertal healthy boys. Although a genome-wide association study (GWAS) revealed that genetic variants of *SULT2A1* do not affect individuals’ DHEA(S) concentrations or the DHEA/DHEAS ratio^35^, such association was never investigated in individuals with ADHD. Therefore, whether genetic variants of *SULT2A1* influence neurosteroid and further correlate to ADHD susceptibility remains unclear.

Evidence has already established that the *STS* and *SULT2A1* genes are vital for catalyzing neurosteroids (DHEA or DHEA-S)^19^,^20^,^33^,^34^, which may participate in ADHD pathophysiology^19^,^27^. However, little is known about the relationship among variants of the *STS* gene and the *SULT2A1* gene, DHEA(S) levels, and ADHD characteristics. Genetic associations for complex diseases may be probed either with case-control studies of unrelated people or with family-based designs^36^,^37^. Relative to case-control designs, family-based study designs (e.g. case-parent trios) have the advantage that there is a common genetic background among the family members and may be applicable for detecting risk genes of ADHD^36^,^37^. To fill in the research gap, this study used a family-based design to investigate the relationships among polymorphisms of the *STS* gene and *SULT2A1* gene, salivary DHEA(S) levels, and ADHD characteristics.

## Methods

### Participants

The study protocol was approved by the Institutional Review Board of Chang Gung Hospital in Taiwan (IRB No: 101-4835A3). All procedures performed in this study involving participants were in accordance with the ethical standards of the institutional and/or national research committee and with the Helsinki Declaration. We obtained written informed consent from the parents or guardians of all the participating children.

We recruited eligible outpatients with ADHD receiving treatment at the Department of Child Psychiatry at Kaohsiung Chang Gung Memorial Hospital in Taiwan. The admissibility criteria for entry into this study were as follows: (1) patients aged between 6 and 16 years who met the criteria for ADHD outlined in the Diagnostic and Statistical Manual of Mental Disorders, 4th Edition (DSM-IV); (2) without a history of neuropsychiatric diseases (such as intellectual disabilities, autism spectrum disorder, bipolar disorders, major depressive disorders, psychotic disorders, substance dependence, epilepsy, or severe head trauma); (3) without any known major physical illnesses (such as genetic, metabolic, or infectious conditions) that require surgical intervention or long-term medication; (4) either newly diagnosed with ADHD or had an existing diagnosis but had not taken medication for ADHD for at least the prior 6 months; (5) of ethnic Han Chinese origin; and (6) with at least one biological parent that can provide the check swab sample and complete this study’s assessment. A total of 208 boys (mean age of 8.7 ± 2.1 years) met the inclusion criteria and agreed to participate in this study.

### Study Procedures

Patients were interviewed by a child psychiatrist using the Chinese version of the Schedule for Affective Disorder and Schizophrenia for School-Age Children, Epidemiologic Version (K-SADS-E). The social and behavioral competence and ADHD symptoms of patients were evaluated with the Swanson, Nolan, and Pelham Questionnaire, Version IV for ADHD (SNAP-IV), which the patients’ parents and teachers completed. The ADHD patients then performed the Wechsler Intelligence Scale for Children-Fourth Edition (WISC-IV), and the Conners’ Continuous Performance Tests (CPT) were performed by an experienced psychologist in a dedicated room for testing so that test condition variability was minimized. Patients’ parents were asked to fill out the 18-question Adult ADHD Self-Report Scale (ASRS).

High molecular weight genomic DNA of ADHD patients and their biological parents were extracted from cheek swabs using routine procedures. The ADHD patients’ saliva samples were collected between 7:00 and 8:00 am using the passive drool method in order to analyze the neuroendocrine substrate levels. Patients were instructed to avoid excessive physical activity during the preceding 24 h and to fast overnight prior to saliva collection.

### Genotyping

The genotyping of three SNPs in the *STS* gene (rs6639786 and rs2270112 in intron 1 and rs17268988 in intron 9) has been carried out in previous literature^29^,^30^,^31^, using custom allelic discrimination TaqMan assays (Applied Biosystems, Foster City, CA, USA). According to genotype data in the HapMap Project (www.hapmap.org), the Han Chinese population has no reported polymorphisms for rs5934770, rs12861247, or rs17335568. Therefore, these SNPs were not considered for further analyses in this study.

The polymorphism rs182420 in the *SULT2A1* gene has been genotyped in previous literature^34^,^35^ using custom allelic discrimination TaqMan assays (Applied Biosystems, Foster City, CA, USA). The allele frequency of rs2637125 was reported to have a great disparity (G/A:0.99/0.01) in the Han Chinese population in the HapMap Project. Therefore, the polymorphism of rs2637125 was also removed from this study. A 7500 Fast RealTime PCR System (Applied Biosystems) was used to make allelic discrimination decisions.

### Laboratory Tests for Neurosteroid Levels

Saliva was put in collecting tubes, immediately placed on ice, and stored at −80°C until assayed. We used an enzyme immunoassay (ELISA) to determine and quantify the DHEA in saliva (IB79306, IBL International GmbH) and also used an ELISA for the *in-vitro* diagnostic quantitative determination of DHEA-S (RE52661/RE52669, IBL International GmbH). The detection range of these methods for DHEA and DHEA-S was 2.2–1440 pg/ml and ≥0.05 ng/mL, respectively. The intra- and inter-assay coefficients of variation were 3.4–4.7% and 3.1–5.6% for DHEA and ≦7.8% and ≦14.9% for DHEA-S.

### Behavioral and Neuropsychological Assessment

The K-SADS-E is a semi-structured diagnostic interview designed to assess current and past episodes of psychopathology in children and adolescents according to DSM-III-R and DSM-IV criteria^38^. The K-SADS-E is administered by interviewing the parent(s) and the child to ultimately determine summary ratings that include all sources of information^39^.

The SNAP-IV is a 26-item questionnaire used to evaluate ADHD symptoms and severity^40^. The 26 items include 18 items related to ADHD symptoms (9 for inattention and 9 for hyperactivity and impulsivity) and 8 pertaining to oppositional defiant disorder symptoms as defined in the DSM-IV-TR. The Chinese version of the SNAP-IV parent form^41^ and the SNAP-IV teacher form^42^ have been reported to have satisfactory levels of reliability and concurrent validity.

The ASRS is a self-administered questionnaire that evaluates ADHD symptoms in adults. The two-part questionnaire consists of inattention and hyperactivity/impulsivity subscales, each of which has nine indications that have persisted for at least 6 months^43^. The validity and reliability of the Chinese version of ASRS have been confirmed through previous studies^44^. Whether the parents had ADHD or not was determined using the ASRS, and the total score of the ASRS > 16 were classified as ADHD cases^45^.

The WISC-IV is an individually administered and norm-referenced tool that aims to measure intelligence in children aged from 6 to 16^46^. The WISC-IV contains 10 core and 5 supplemental subtests. The core subtests are used to form four factor indexes, including the Verbal Comprehension Index (VCI), the Perceptual Reasoning Index (PRI), the Working Memory Index (WMI), and the Processing Speed Index (PSI). The Chinese version of the WISC-IV has been validated by prior studies^47^.

The CPT is a 14-minute computerized test that mainly judges attention and impulse control^48^. In short, participants have to respond to the stimuli on a computer screen by pressing a space bar for every letter except the letter “X.” The test includes various assessment parameters, including Omissions, Commissions, Hit Reaction Time (RT), Hit Reaction Time Standard Error (Hit RT SE), Variability of Standard Error, and Detectability (D’). The T-score of each index was used for research analyses, and a lower T-score represents a better performance. Finally, a Confidence Index (percentile) integrates all the CPT data obtained from the test’s administration to provide a probability out of 100 that a significant attention problem exists^49^.

### Statistical Analyses

Data were analyzed using the Statistical Package for the Social Sciences for Windows (version 16.0; SPSS, Inc., Chicago, IL, USA). Variables are presented as either mean ± standard deviation (SD) or frequency.

The transmission disequilibrium test (TDT) was originally developed to test for the transmission of associated marker locus by studying case-parent trios from a heterozygous parent to an affected child^50^. In this study, TDT analysis was conducted using PLINK v1.90b3.38 (7 Jun 2016) and was applied to the collection of trio data in this study. Odds ratio, chi-square statistic, and *p*-value were calculated for each allele of interest.

Because data on the DHEA and DHEA-S levels exhibited significant positive skews and violated normal distribution, and the case numbers of the minor allele carriers were small, non-parametric statistics were used to analyze the data. Genotypes of the SNPs (rs6639786, rs2270112, and rs17268988) within *STS* gene were classified based on the allele identified on X-chromosome, and genotypes of rs182420 in the *SULT2A1* gene were classified into C/T or T/T. Metric variables between genotypes were tested using the t-test or the Mann-Whitney U test. The Mann-Whitney U test was used to determine the potential difference in neurosteroid levels between patients with different genotypes. Spearman’s correlation coefficient was used to investigate the relationship between neurosteroid levels and ADHD characteristics. Two-tailed *p*-values < 0.05 were considered statistically significant. Bonferroni correction was further performed to adjust for multiple testing in the correlation matrix.

## Results

Of the 208 ADHD boys recruited in this study, 200 patients provided their genomic DNA with a cheek swab; the saliva samples of 160 patients were successfully collected; and 173 patients completed the clinical and neuropsychological assessments. Table 1 lists the characteristics of the 200 boys whose genomic DNA data were available (mean ages of 8.7 ± 2.1 year). We found that C allele carriers of rs2270112 had significantly lower SNAP-IV oppositional scores rated by teachers (p = 0.048) and higher detectability T-scores (p = 0.015) than G allele carriers. Additionally, patients with rs182420 C/T genotype within the *SULT2A1* gene had higher variability scores (p = 0.006) than those with T/T genotype.

Of the 200 boys with ADHD with genomic DNA data, both parents were available for 150 families, while only one biological parent was available for the remaining 50. Genotyping was successful for over 90% of the samples in all assays. We found that the C allele of rs2270112 within the *STS* gene (p = 0.020) was over-transmitted in males with ADHD. The other two SNP markers located in the *STS* gene (rs6639786 and rs17268988) and rs182420 in the *SULT2A1* gene were not associated with ADHD (Table 2).

**Figure 1:**
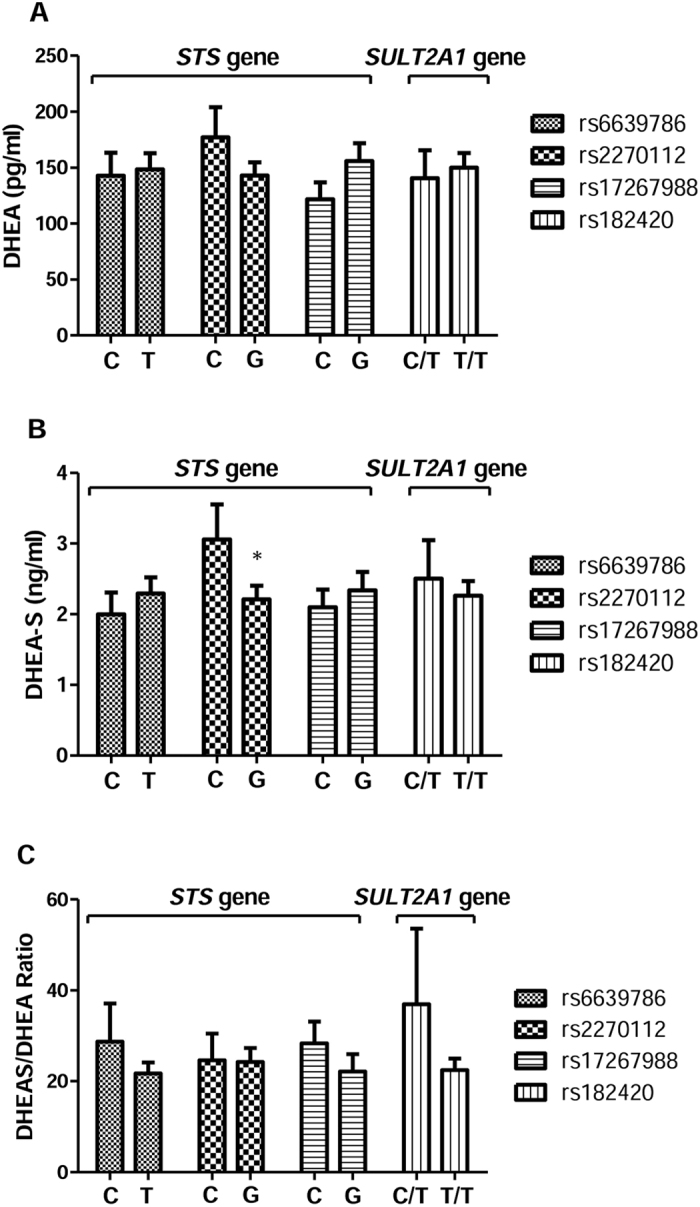
The salivary levels of (**A**) dehydroepiandrosterone (DHEA), (**B**) dehydroepiandrosterone sulfate (DHEA-S) and (**C**) DHEA-S/DHEA ratio between patients with different genotypes of the *STS* gene and the *SULT2A1* gene. *p < 0.05.

The salivary DHEA, DHEA-S levels and DHEA-S/DHEA ratios between polymorphisms of the *STS* gene and *SULT2A1* gene are shown in Fig. 1. The C allele carrier of rs2270112 had significantly higher DHEA-S levels (mean difference = 0.85 ng/ml, p = 0.014) than the G allele carrier. However, salivary DHEA levels and DHEA-S/DHEA ratios were not significantly different between patients with various genotypes.

The relationships of salivary neurosteroid levels and ADHD characteristics are provided in Table 3. Salivary DHEA levels showed a significant negative correlation with many indices of CPT, including Confidence Index (r = −0.25, p = 0.002), Omission (r = −0.23, p = 0.005), Hit RT (r = −0.23, p = 0.004), Hit RT SE (r = −0.26, p = 0.001), and Variability (r = −0.22, p = 0.008). If we used Bonferroni correction to adjust for multiple testing in the correlation matrix (*p*-value = 0.05/13 = 0.0038), DHEA levels were only significantly correlated to Confidence Index and Hit RT SE. We observed no significant correlations between salivary DHEA(S) levels and behavioural symptoms.

## Discussion

Our results reveal that rs2270112 within the *STS* gene was over-transmitted in Han Chinese boys with ADHD. Polymorphisms of rs182420 within the *SULT2A1* gene were not associated with ADHD. Since the C allele of rs2270112 within the *STS* gene was over-transmitted in males with ADHD, we explored whether rs2270112 polymorphisms influence *STS* gene function, reflected by patients’ nerurosteroids levels. Among boys with ADHD, the C allele carriers of rs2270112 had significantly higher DHEA-S levels than the G allele carriers. In addition, we found that the salivary levels of DHEA were correlated with several neuropsychological indices measured by the CPT.

To the best of our knowledge, this study is the first to investigate the genetic variants of the *STS* gene and the *SULT2A1* gene in ADHD among a non-Caucasian population. A previous study using family-based design^30^ indicated that as many as seven polymorphisms within the *STS* gene were significantly associated with ADHD. Furthermore, another study using case-control design^31^ found three SNPs within the *STS* gene were associated with cognitive function in ADHD males. In contrast, we found that only one SNP (rs2270112) of the *STS* gene was over-transmitted in ADHD boys. The inconsistent findings may be attributed to discrepancies of sample size, study designs, or possible ethnic differences in genetic variants. For example, several SNPs of the *STS* gene that have been associated with Caucasian ADHD patients (rs5934770, rs12861247, and rs17335568) do not exhibit polymorphisms in the Han Chinese population. Additionally, it has been reported that the G allele at rs17268988 is the minor allele^51^, whereas we found the C allele is the minor allele of this SNP among our study population. Therefore, the findings of previous genetic studies conducted in the Caucasian population may not be universally applied across ethnicities. However, these results support the hypothesis of previous literature that ADHD susceptibility is associated with variants or functions of the *STS* gene in males with ADHD.

We found that the C allele of rs2270112 at *STS* gene was significantly associated with higher DHEA-S levels in boys with ADHD. The *STS* protein is a microsomal enzyme that converts DHEA-S into its unsulfated form of DHEA, which participates in a neurosteroid biosynthesis pathway of the brain^5^. Current evidence related to how *STS* genetic variants affect DHEA(S) concentrations is still scarce^26^,^52^,^53^. Relative to previous investigations, the current study is advantaged in providing data about DHEA, DHEA-S and DHEA-S/DHEA ratios specific in ADHD population. In addition, we performed comprehensive assessments for patients’ behavioural symptoms and neuropsychological function (CPT). By demonstrating the association between the rs2270112 within the *STS* gene with the synthesis or transformation of DHEA(S) levels in patients with ADHD, our result suggest the rs2270112 may be linked to the neurobiology pathogenesis of ADHD^8^,^54^.

Our study’s results reveal that the polymorphisms of rs182420 in the *SULT2A1* gene were not associated with ADHD, and did not influence the salivary DHEA or DHEA-S levels in ADHD boys. However, Garcia-Anguita *et al*.^34^ indicated that 792 healthy post-pubertal (12–16-year-old) boys homozygous for the allele of rs182420 (TT) showed higher DHEA-S concentration than heterozygous TC boys, followed by CC boys. These contradictory findings may be to the result of ethnic differences, discrepancies in target populations, or sample sizes. For example, no participants carried a CC genotype of rs182420 in our study population, which indicates that the variants distribution of the *SULT2A1* gene may differ between ethnicities. The *SULT2A1* gene has been reported as a catalyst for the sulfate conjugation of DHEA^33^. Nevertheless, data regarding whether the genotypes of *SULT2A1* appear to have effects on individuals’ DHEA(S) have been inconsistent in previous studies^34^,^35^. Our data did not support that *SULT2A1* gene plays a role in the susceptibility of ADHD.

The results of this study show that several indexes measured by CPT were positively correlated with salivary DHEA levels, particularly Confidence Index and Hit RT SE. These findings are in line with our previous investigations in which an independent cohort was recruited^10^,^13^. The Confidence Index represents the probability that an overall attention problem exists, and the Hit RT SE indicates the consistency of response times for responses to target^48^,^49^. In animal studies, 39,X(Y)*O mice exhibit deficits in the performance of 5-choice serial reaction time task (5-CSRTT)^55^, whereas DHEA-S administration could reverse these deficits under attentional demanding conditions^22^. The 5-CSRTT, a rodent analogue of the human CPT, is a laboratory behavioral task for assessing neurobiological substrates of attention^56^. Therefore, compatible with our findings, previous data in mice have suggested a link between attention and DHEA(S) levels^9^,^21^,^22^,^23^,^24^. The protective effects of DHEA(S) on patients’ neuropsychological function may account for enhancing neuron growth^5^, regulating GABA_A_ and NMDA activity^6^,^7^, or modulating the complex process of cortical maturation during middle childhood^57^,^58^. DHEA and DHEA-S may also play independent roles in some aspects of the nervous system. For example, DHEA-S is better at improving neuronal survival^59^ and stimulating catecholamine synthesis and secretion^60^; in contrast, DHEA outperforms DHEA-S in neurogenesis^61^. We proposed that DHEA may exert certain neurobiological functions among boys with ADHD. Nevertheless, although the CPT provides an objective assessment for attention^62^, the neurocognitive deficits measured by the CPT may not be specific to ADHD^63^. Whether the relationships between DHEA levels and CPT performance were specific for ADHD patients warrants further investigation.

The subjects recruited in our study were all males. Compelling evidence have revealed that male and female ADHD patients have distinct clinical manifestations^64^,^65^, neuropsychological functions and responses to medication treatment^66^,^67^,^68^. Various neurobiological domains have been proposed to underlie the sex differences in attention and impulsivity, including neurosteroids and their associated genes^69^,^70^. Future studies are required to explore whether *STS* and *SULT2A1* genes also play a role in female ADHD patients.

This study has several limitations. First, the case number was small, and that reduced statistic power lowers the likelihood of finding a potential association between genetic variants and ADHD. Second, the target SNPs of the *STS* gene and *SULT2A1* gene in this study were chosen based on the findings of previous literature. However, several SNPs that have been found to be associated with ADHD in Caucasian populations did not exhibit polymorphisms in our Han-Chinese study sample. Therefore, advanced techniques, such as next-generation sequencing, may be more beneficial for discovering the genetic variants associated with ADHD^71^. Third, the diagnoses of ADHD for the parents were determined using the ASRS, lacking validation using structural diagnostic instruments. Thus, it is possible that some patients’ parents were misclassified as either case or control. Therefore, the current study result should be interpreted with caution. Fourth, multiple testing correction was not performed in the statistical analyses. If Bonferroni correction had been performed to adjust for multiple testing in the correlation matrix (Tables 2 and 3), few of our significant findings would remain. Finally, we used saliva samples to measure the neurosteroid levels in this study. Although a previous study demonstrated that hormone concentrations in plasma, urine, saliva, and hair are highly correlated^72^, the DHEA(S) levels measured in saliva may not necessarily represent the concentration and activity of neurosteroids in the brain.

In conclusion, our findings suggest that *STS* gene polymorphisms and DHEA(S) levels may related to ADHD susceptibility. Among Han Chinese boys with ADHD, polymorphisms of rs2270112 within the *STS* gene were over-transmitted, and are associated with DHEA-S concentration. Salivary levels of DHEA were positively correlated with attention measured by a neuropsychological test. Future research with a considerably larger sample size is needed to determine how neurosteroids and their associated genes are involved in the underlying biological mechanisms of ADHD.

## Additional Information

**How to cite this article:** Wang, L.-J. *et al*. Polymorphisms of *STS* gene and *SULT2A1* gene and neurosteroid levels in Han Chinese boys with attention-deficit/hyperactivity disorder: an exploratory investigation. *Sci. Rep.* **7**, 45595; doi: 10.1038/srep45595 (2017).

**Publisher's note:** Springer Nature remains neutral with regard to jurisdictional claims in published maps and institutional affiliations.

## Figures and Tables

**Table 1:** Characteristics of boys with attention-deficit/hyperactivity disorder, and comparisons of different genotypes.

Characteristics	Total	*STS* gene rs6639786		*STS* gene rs2270112		*STS* gene rs17268988		*SULT2A1* gene rs182420	
	(N = 200)	C allele (n = 50)	T allele (n = 130)	C allele (n = 19)	G allele (n = 179)	C allele (n = 77)	G allele (n = 110)	C/T (n = 22)	T/T (n = 163)
**Age** (year)	8.7 ± 2.1	8.8 ± 2.2	8.7 ± 2.1	8.8 ± 2.1	8.7 ± 2.1	8.6 ± 2.1	8.7 ± 2.2	8.1 ± 1.4	8.9 ± 2.2
**WISC-IV**									
FSIQ	95.0 ± 12.6	94.3 ± 13.0	95.4 ± 12.6	98.3 ± 7.6	94.6 ± 12.9	96.2 ± 11.8	94.3 ± 12.8	96.9 ± 13.0	94.7 ± 12.4
VCI	98.9 ± 12.4	100.0 ± 13.5	98.6 ± 11.9	99.1 ± 9.4	98.8 ± 12.5	99.0 ± 12.9	99.5 ± 12.1	100.2 ± 12.1	98.6 ± 11.9
PRI	95.8 ± 15.2	92.4 ± 16.0	97.5 ± 15.1	98.0 ± 5.8	95.3 ± 15.7	97.9 ± 14.1	93.9 ± 15.2	99.9 ± 16.0	95.8 ± 15.2
WMI	95.9 ± 13.6	93.3 ± 13.2	97.2 ± 14.3	98.5 ± 10.9	95.6 ± 14.1	96.8 ± 14.7	95.6 ± 13.3	98.0 ± 14.1	96.0 ± 13.8
PSI	93.3 ± 14.3	95.1 ± 12.3	92.0 ± 14.7	99.4 ± 13.6	92.7 ± 14.0	93.3 ± 12.0	92.9 ± 14.9	90.8 ± 14.1	92.7 ± 13.5
**Clinical measures**									
SNAP-IV parent form (I)	14.6 ± 5.5	14.8 ± 5.4	14.8 ± 5.7	13.6 ± 6.0	14.9 ± 5.5	13.9 ± 5.5	15.3 ± 5.2	15.1 ± 5.5	14.6 ± 5.6
SNAP-IV parent form (H)	12.9 ± 6.0	13.4 ± 5.6	12.6 ± 6.3	11.4 ± 5.5	13.1 ± 6.0	12.8 ± 5.4	12.9 ± 6.3	13.6 ± 5.5	12.7 ± 6.1
SNAP-IV parent form (O)	10.7 ± 5.8	11.0 ± 5.3	10.3 ± 5.9	8.3 ± 5.4	10.8 ± 5.8	10.8 ± 5.8	10.1 ± 5.5	11.0 ± 6.3	10.7 ± 5.8
SNAP-IV teacher form (I)	14.6 ± 6.2	13.6 ± 5.9	14.5 ± 6.4	11.9 ± 6.8	14.7 ± 6.2	15.4 ± 6.3	14.0 ± 6.1	13.2 ± 6.1	14.6 ± 6.1
SNAP-IV teacher form (H)	11.9 ± 7.4	11.7 ± 6.8	11.9 ± 7.6	9.6 ± 8.9	12.1 ± 7.2	12.3 ± 7.3	11.8 ± 7.4	12.2 ± 6.2	12.1 ± 7.5
SNAP-IV teacher form (O)	7.7 ± 6.3	8.0 ± 5.7	7.5 ± 6.4	4.6 ± 4.9	7.9 ± 6.2*^a^	7.9 ± 6.4	7.3 ± 6.0	6.9 ± 6.1	7.9 ± 6.4
**Index of CPT**									
Confidence Index (%)	61.2 ± 22.3	59.9 ± 21.0	62.5 ± 23.0	66.5 ± 26.0	61.1 ± 22.3	61.6 ± 21.3	62.7 ± 23.7	68.1 ± 22.2	60.3 ± 22.6
Omission	62.0 ± 29.9	59.2 ± 30.4	64.1 ± 31.4	72.7 ± 44.8	61.2 ± 27.9	58.2 ± 20.4	65.6 ± 35.5	69.8 ± 41.2	61.5 ± 29.3
Commission	49.9 ± 10.6	50.0 ± 11.0	50.0 ± 10.7	49.9 ± 10.4	49.8 ± 10.7	50.9 ± 9.9	48.9 ± 11.2	51.2 ± 9.8	49.7 ± 10.8
Hit Reaction Time	55.6 ± 12.9	55.8 ± 12.5	55.8 ± 13.1	57.2 ± 14.5	55.7 ± 12.6	55.0 ± 10.7	57.0 ± 14.4	55.7 ± 14.2	55.4 ± 12.8
Hit Reaction Time SE	58.0 ± 12.0	57.6 ± 11.7	58.8 ± 11.9	61.5 ± 14.0	58.0 ± 11.6	58.3 ± 10.2	58.7 ± 13.1	62.2 ± 12.6	57.5 ± 12.0
Variability	58.1 ± 11.3	56.6 ± 10.6	59.2 ± 11.2	61.7 ± 11.7	57.9 ± 11.1	58.1 ± 10.7	58.7 ± 11.7	64.6 ± 9.6*^c^	57.4 ± 11.3
Detectability	51.6 ± 9.8	51.9 ± 10.8	51.6 ± 9.4	56.6 ± 8.3*^b^	50.9 ± 9.8	51.3 ± 9.0	51.5 ± 10.7	52.2 ± 7.7	51.4 ± 9.8

Note: data are expressed as N (%) or Mean ± SD; WISC-IV, the Wechsler Intelligence Scale for Children–Fourth Edition; FSIQ, Full Scale Intelligence Quotient; VCI, Verbal Comprehension Index; PRI, Perceptual Reasoning Index; WMI, Working Memory Index; PSI, Processing Speed Index; SNAP-IV, the Swanson, Nolan, and Pelham–Version IV Scale for ADHD; CPT, Conners’ Continuous Performance Test; I, inattention scores; H, hyperactivity/impulsivity scores; O, oppositional scores. SE, Standard Error. *p < 0.05 under comparison. ^a^*Z* = 1.980, *p* = 0.048; ^b^*Z* = 2.425, *p* = 0.015; ^c^*Z* = 2.733, *p* = 0.006.

**Table 2:** Transmission disequilibrium test for polymorphisms of the *STS* gene and the *SULT2A1* gene among boys with ADHD and their parents.

Gene	SNP	BP	A1	A2	T	U	OR	χ^2^	*p*-value
*STS (CHR X*)	rs6639786	7161475	C	T	18	14	1.188	0.500	0.4795
*STS (CHR X*)	rs2270112	7170925	C	G	3	12	0.429	5.400	0.0201*
*STS (CHR X*)	rs17268988	7254481	C	G	16	11	1.200	0.926	0.3359
*SULT2A1 (CHR 19*)	rs182420	48372195	C	T	12	20	0.565	2.000	0.1573

Note: BP, base pair; OR, odds ratio. Transmitted (T) and Untransmitted (U) minor allele count. *p < 0.05.

**Table 3:** Correlations between levels of neuroendocrine substrates and measures of ADHD.

ADHD variables	DHEA		DHEA-S	
	*r*	*p*-value	*r*	*p*-value
**Clinical measures**				
SNAP-IV parent form (I)	0.057	0.474	0.136	0.087
SNAP-IV parent form (H)	−0.108	0.175	−0.080	0.314
SNAP-IV parent form (O)	0.133	0.093	0.142	0.073
SNAP-IV teacher form (I)	−0.067	0.406	0.014	0.866
SNAP-IV teacher form (H)	−0.067	0.407	−0.044	0.586
SNAP-IV teacher form (O)	−0.022	0.788	0.064	0.429
**Index of CPT**				
Confidence Index (%)	−0.259	0.002*	−0.114	0.168
Omission	−0.231	0.005	−0.059	0.477
Commission	−0.010	0.895	0.026	0.756
Hit Reaction Time	−0.233	0.004	−0.145	0.079
Hit Reaction Time SE	−0.291	0.001*	−0.161	0.052
Variability	−0.228	0.008	−0.087	0.297
Detectability	−0.056	0.542	−0.009	0.914

Note: Data are expressed using Correlation Coefficient; DHEA, dehydroepiandrosterone; DHEA-S, dehydroepiandrosterone sulfate; SNAP-IV, the Swanson, Nolan, and Pelham–Version IV Scale for ADHD; CPT, Conners’ Continuous Performance Test; I, inattention scores; H, hyperactivity/impulsivity scores; O, oppositional scores; SE, Standard Error. If we used Bonferroni correction to adjust for multiple testing in the correlation matrix (*p*-value = 0.05/13 = 0.0038), DHEA levels were significantly correlated to Confidence Index and Hit Reaction Time SE. *Significant correlation remains after Bonferroni correction.
